# Peptide‐Drug Conjugates: A New Hope for Cancer

**DOI:** 10.1002/psc.70040

**Published:** 2025-07-11

**Authors:** Amy Armstrong, Fleur Coburn, Yanyamba Nsereko, Othman Al Musaimi

**Affiliations:** ^1^ School of Pharmacy Newcastle University Newcastle upon Tyne UK; ^2^ Department of Chemical Engineering Imperial College London London UK; ^3^ Orthogonal Peptides Limited London UK

**Keywords:** cancer, cleavable and noncleavable linkers, PDC, peptides, receptor‐less‐targeting, targeted therapy

## Abstract

Peptide‐drug conjugates (PDCs) are advancing as targeted cancer therapies, leveraging lessons from antibody‐drug conjugates (ADCs) to improve tumour specificity. These molecules combine a homing peptide with a cytotoxic payload via a linker, enabling precise drug delivery while sparing healthy tissue. Despite their potential, PDCs face challenges including metabolic instability, premature payload release and rapid clearance, limiting clinical success. Only Lutathera remains FDA‐approved after Pepaxto's withdrawal, though Pepaxto retains EMA and MHRA approval—highlighting regulatory and technical complexities. Most PDCs target overexpressed receptors (e.g., somatostatin and GnRH), though novel designs like CBX‐12 employ alternative strategies. Currently, six PDCs are in Phase III trials, with ~96 in development, signalling growing interest. This review explores how ADC research has guided PDC optimisation, particularly in linker chemistry and payload selection. We analyse key structural features governing PDC efficacy, including peptide‐receptor binding and intracellular trafficking. Innovations in stable linkers and tumour‐selective activation mechanisms are critical to overcoming pharmacokinetic hurdles. Promising candidates in late‐stage trials are highlighted, emphasising their potential to address unmet needs in oncology. By refining targeting precision and payload delivery, next‐generation PDCs may expand treatment options for resistant cancers, bridging the gap between biologics and small‐molecule therapies.

## Graphical abstract

Peptide‐drug conjugates (PDCs) are emerging cancer therapeutics inspired by antibody‐drug conjugates, offering targeted delivery via peptide ligands. Despite structural versatility, challenges like instability and rapid clearance hinder clinical progress. Innovations in linker design and targeting strategies drive ongoing development, with several candidates advancing through late‐stage clinical trials.

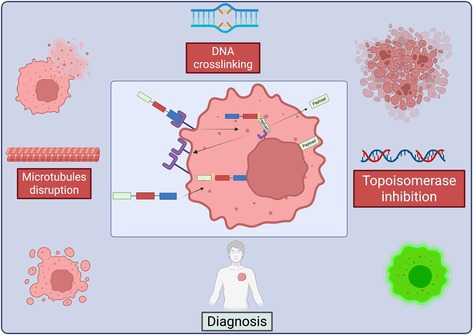

## 1 Introduction

Cancer is characterised by uncontrolled cellular proliferation and aberrant growth, leading to invasive spread and destruction of healthy tissues [1]. As one of the leading causes of death globally, it is projected to claim 10 million lives by 2030 [2, 3]. The current standard of care primarily involves surgery followed by chemotherapy, but the lack of tumour‐specific targeting results in severe off‐target toxicity, limiting its clinical utility and contributing to a burdensome side‐effect profile [4]. Beyond its physiological toll, chemotherapy imposes significant economic and societal costs—including high treatment expenses, lost productivity (with ~50,000 working‐age deaths annually) and profound psychosocial strain on patients and their families [5]. The demanding treatment regimen often leads to social isolation, further compounding the challenges faced by cancer patients.

Peptide‐based therapeutics have gained increasing clinical relevance, with the FDA approving 33 peptide drugs between 2016 and 2024 [6, 7]. Notable examples include trofinetide (Daybue) for Rett syndrome and levacetylleucine (Aqneursa) for Niemann–Pick disease Type C, highlighting peptides' potential in treating rare genetic disorders [6, 8, 9]. In oncology, peptides offer transformative advantages over conventional therapies, including exceptional target specificity, the ability to disrupt oncogenic protein–protein interactions (PPIs) and minimal off‐target effects [10]. Their high tumour penetration, biocompatibility and adaptability to advanced drug delivery systems (e.g., ADCs) further enhance their therapeutic appeal [11]. The growing success of peptide‐based drugs underscores their expanding role in precision medicine and supports their continued development for cancer treatment.

**FIGURE 1:**
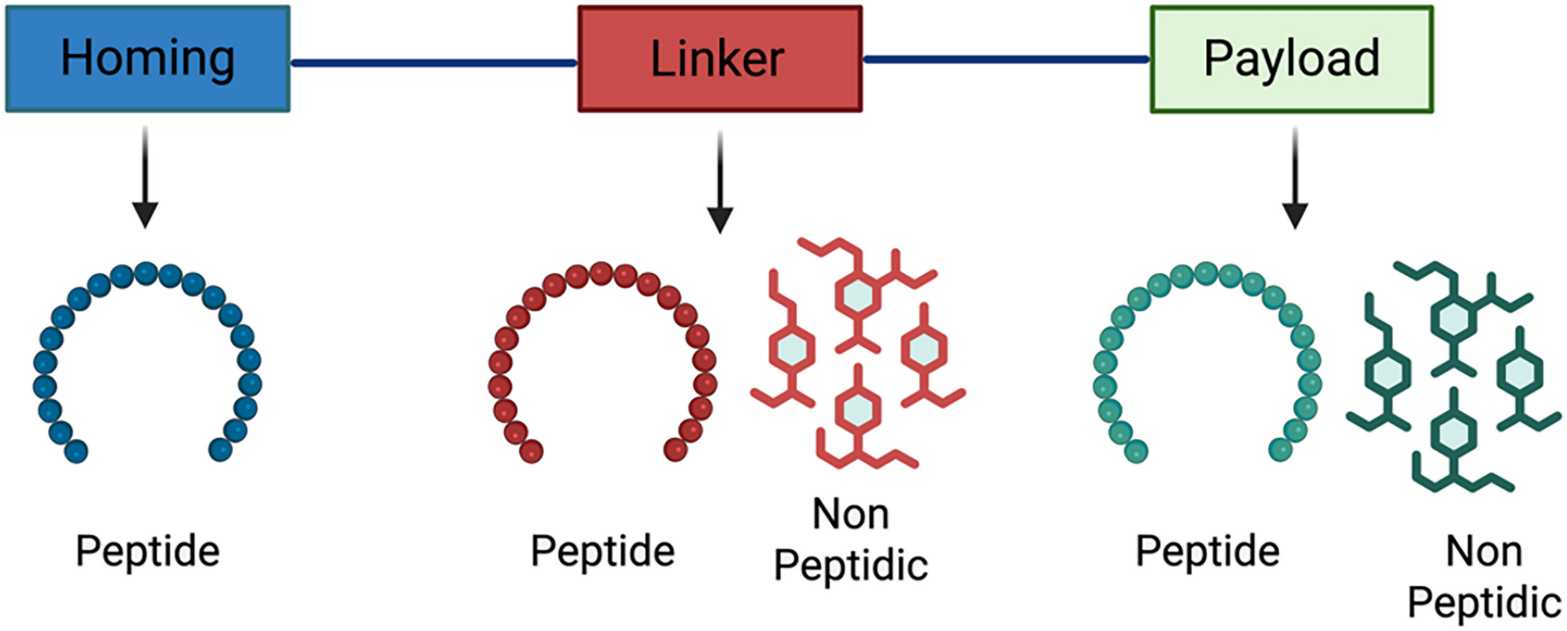
Basic structure of PDC. Created using www.BioRender.com.

PDCs exemplify this progress, combining a tumour‐targeting peptide (TTP), a cytotoxic payload and a linker molecule into a single therapeutic agent (Figure 1). This modular design enables selective drug delivery to cancer cells, maximising efficacy while minimising systemic toxicity [2]. As PDC technology evolves, innovations in linker stability and payload release mechanisms are expected to further improve their clinical performance, positioning them as a promising frontier in targeted cancer therapy.

Peptides possess a unique dual functionality, enabling their use in both diagnostic and therapeutic applications—a concept termed theranostics. Lutathera exemplifies this approach as the only FDA‐approved PDC currently on the market that fully integrates theranostic capabilities [12].

Peptide receptor radionuclide therapy (PRRT) structurally aligns with the definition of a PDC, comprising a targeting peptide, a linker (often a chelator such as DOTA) and a payload—albeit a radionuclide (e.g., Lutetium‐177 or Yttrium‐90) rather than a conventional cytotoxic drug [13]. Despite this similarity, PRRT is frequently classified separately because of its roots in nuclear medicine, its reliance on radiation‐induced tumour killing (as opposed to chemical cytotoxicity) and its distinct regulatory framework [3]. Although PDCs traditionally refer to chemotherapeutic conjugates, the broader definition may encompass PRRT, particularly as emerging hybrid therapies combine radionuclides with drug payloads. Thus, PRRT can be considered a specialised subclass of PDCs—one that employs radiotherapy as its primary mechanism of action.

This review critically evaluates the scientific landscape of PDCs, assessing their therapeutic advantages, clinical progress and persisting challenges. By analysing current research, we provide a balanced perspective on their potential to redefine targeted cancer therapy.

## 2 ADCs: Foundations and Limitations

ADCs represent a paradigm shift in targeted cancer therapy, comprising a monoclonal antibody (mAb) linked to a cytotoxic payload via a stable linker. First conceptualised by Paul Ehrlich in the 1950s as ‘magic bullets’, ADCs achieved clinical approval in 2000, marking a milestone in selective cancer treatment [14]. By exploiting tumour‐associated antigen overexpression, ADCs deliver cytotoxic agents with enhanced specificity, minimising off‐target effects [15]. This targeting principle has been instrumental not only for ADC efficacy but also for inspiring PDC development, which further refines tumour homing through smaller, more penetrative peptide moieties [16].

Critical to ADC success are optimised linker systems that balance stability and selective payload release [16]. Recent innovations include hydrophilic, glutamate‐containing linkers (EEVC/EVC), which address aggregation and premature cleavage issues associated with conventional Val‐Cit linkers [17]. These advancements enable higher drug‐to‐antibody ratios (DARs) while resisting enzymatic degradation—key improvements for next‐generation ADCs [17]. Additionally, multifunctional linkers now support dual‐payload delivery, overcoming resistance mechanisms that plague single‐drug ADCs [18, 19]. Song and colleagues recently developed a size‐exclusion chromatography method to accurately assess critical ADC quality attributes, including DAR, free‐drug‐related impurities (FDRI) and purity—key parameters for therapeutic efficacy and safety [20].

Despite their clinical impact—with 10 FDA‐approved ADCs including recent additions like datopotamab deruxtecan [6]—ADCs face persistent challenges: antigen resistance, tumour penetration limitations and linker instability [16, 21, 22, 23]. Furthermore, a major limitation is the development of antigen resistance, which impedes ADC efficacy through multiple mechanisms: target antigen downregulation, activation of compensatory signalling pathways and efflux of cytotoxic payloads via ATP‐binding cassette (ABC) transporters [16]. These hurdles underscore the need for alternative platforms, prompting the exploration of peptides as versatile components for next‐generation conjugates [24].

## 3 Peptides: Bridging ADCs to PDCs

Peptides (2–50 amino acids; 2–20 kDa) are biologically compelling because of their roles in signalling, immunity and hormonal regulation [6, 25]. Their modularity allows precise engineering for therapeutic applications, notably in PDCs, which leverage peptides' superior pharmacokinetics and tumour penetration over antibodies [11]. Although ADCs and PDCs share targeting strategies (Table 1), PDCs address ADC limitations through smaller size, reduced immunogenicity and enhanced tissue diffusion—positioning them as a transformative evolution in targeted therapy [16].

**TABLE 1:** Comparison between ADC and PDC [20, 26, 27].

Property	PDC	ADC
Structure	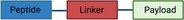	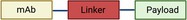
Cost	Low and simple	High and complex
Immunogenicity	Low	High
Clearance	Eliminated by the kidney, fast	Metabolised by the liver, slow
Molecular weight	Small (2–20 kDa), making tissue penetration easier	Large (> 150 kDa), limiting tissue penetration
Half life	Short	Long
Pharmacokinetics	Simple due to production of single homogenous entities	Complex due to heterogenous mixture formation
Specificity for its target	High specificity towards respective receptors	High specificity towards their antigens
Drug loading potency	High drug‐to‐peptide ratios	Low DARs

Abbreviations: DARs, drug‐to‐antibody ratios; mAb, monoclonal antibody.

## 4 Targeting Mechanisms of PDC Homing Peptides

The homing peptide component of PDCs serves as a critical targeting moiety, enabling precise cancer cell recognition through selective binding to overexpressed receptors or tumour‐specific antigens [2]. This molecular targeting strategy significantly reduces off‐target toxicity while enhancing drug delivery to malignant tissues [28]. PDCs exploit distinct tumour biomarkers—such as somatostatin receptors in neuroendocrine tumours (NETs) or prostate‐specific membrane antigen (PSMA) in prostate cancer—to achieve exceptional therapeutic specificity [29]. By capitalising on these molecular differences between cancerous and healthy cells, PDCs demonstrate improved efficacy and safety profiles compared with conventional therapies [2, 29].

**FIGURE 2:**
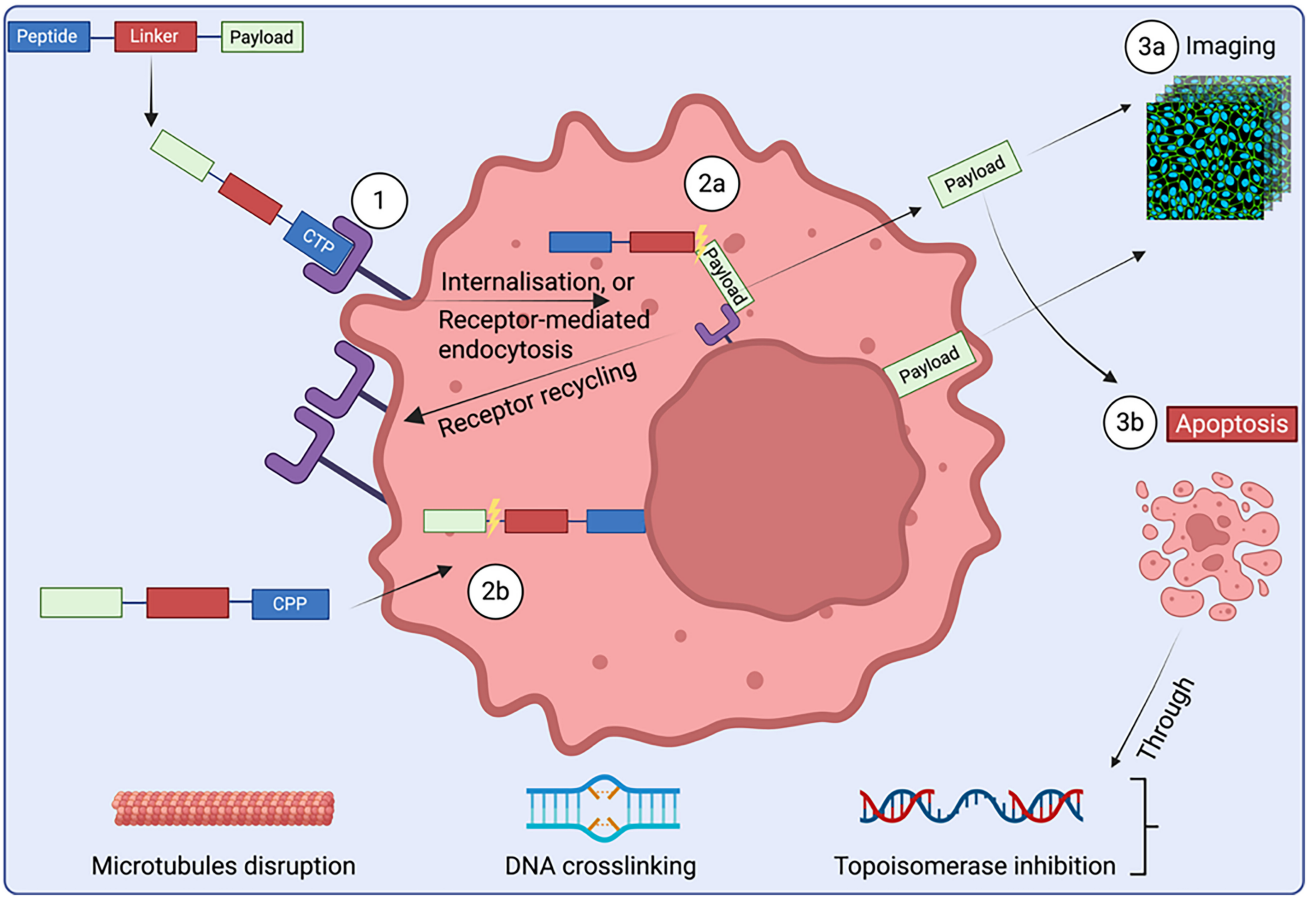
Schematic illustration of PDC mechanism of action: Step 1, CTP binding to overexpressed receptor; Step 2a, CTP linker cleavage (⚡️); Step 2b, CPP linker cleavage (⚡️); Step 3a, cancer cell diagnosis; Step 3b, cancer cell apoptosis. Created using www.BioRender.com.

Homing peptides are broadly classified into two functional categories: (i) cell‐targeting peptides (CTPs), which bind specifically to overexpressed surface receptors, enabling localised drug delivery at high concentrations, and (ii) cell‐penetrating peptides (CPPs), which facilitate intracellular transport through either endocytic pathways or direct membrane translocation while maintaining plasma membrane integrity (Figure 2) [2].

The synergistic combination of CTP specificity with tumour microenvironment (TME)–responsive structural modifications represents a particularly promising approach. The TME can induce helical conformations in homing peptides, a structural transformation that enhances membrane interactions and targeting efficiency [30]. This helical conformation plays a pivotal role in (i) optimising receptor binding affinity, (ii) facilitating membrane penetration, (iii) improving tumour tissue retention and (iv) enhancing payload delivery precision [30]. The strategic integration of these targeting mechanisms positions PDCs as a next‐generation platform for precision oncology, addressing many limitations of current targeted therapies while maintaining favourable pharmacokinetic profiles.

The strategic selection of linker chemistry represents a critical determinant of PDC efficacy, requiring careful optimisation of two competing properties: (1) sufficient plasma stability to prevent premature payload release during systemic circulation and (2) efficient cleavage at the target site to ensure maximal therapeutic activity (see Section 3 for detailed mechanisms). This delicate balance directly impacts both the therapeutic index and safety profile of PDC constructs.

Upon successful tumour targeting and intracellular processing, the liberated payload exerts its cytotoxic effect through apoptosis induction (Figure 2). Notably, many highly potent cytotoxic agents demonstrate limited clinical utility as free drugs due to suboptimal pharmacokinetic profiles, dose‐limiting systemic toxicity or narrow therapeutic windows. The PDC platform overcomes these limitations through targeted delivery, enabling reduced effective doses and therefore enhancing safety, increased tumour‐specific accumulation and minimised exposure to healthy tissues [2]. This paradigm shifts from systemic to targeted chemotherapy capitalises on the differential expression of tumour biomarkers, effectively transforming otherwise undruggable cytotoxic agents into precision therapeutics [2]. The conjugation strategy not only mitigates traditional chemotherapy side effects but also potentiates tumour‐selective cytotoxicity through receptor‐mediated internalisation and intracellular payload release.

## 5 PDC Targeting Paradigms

### 5.1 Receptor‐Independent Homing Peptide Paradigm

PDCs offer a unique advantage in oncology through their dual targeting capabilities. Unlike traditional receptor‐dependent delivery systems, certain PDCs can bypass receptor‐mediated endocytosis pathways and achieve intracellular payload delivery independent of surface receptor expression [31]. This receptor‐independent mechanism provides critical benefits, including overcoming limitations of receptor downregulation in cancer cells, enabling delivery to tumours with heterogeneous antigen expression, expanding the range of targetable malignancies and maintaining efficacy in receptor‐poor TMEs. The membrane‐permeabilizing capacity of certain peptides represents a paradigm shift in drug delivery, complementing traditional receptor‐targeted approaches and addressing a key limitation of antibody‐based delivery systems. This multimodal targeting strategy significantly enhances the therapeutic potential of PDCs across diverse cancer types [31, 32].

#### 5.1.1 Pepaxto or Pepaxti (Melphalan Flufenamide)

Pepaxto, a PDC developed by oncopeptides, received FDA‐accelerated approval in February 2021 for relapsed/refractory multiple myeloma (combined with dexamethasone) [2]. Its design leveraged aminopeptidase‐mediated cleavage to deliver an alkylating payload directly into tumour cells, bypassing receptor dependence. However, postapproval trials failed to confirm clinical benefit, instead revealing increased mortality risk. The FDA mandated withdrawal in October 2021—a rare reversal underscoring the challenges of accelerated approval mechanisms when confirmatory data contradict early findings [33].

For PDCs to progress towards clinical approval, rigorous research must focus on overcoming their key limitations. A primary challenge is improving stability by addressing issues such as premature cleavage [34] and rapid renal clearance [35], which could be tackled through advanced linker technologies and structural modifications. In addition, a deeper investigation into the safety decline observed with Pepaxto [33] is crucial to identify underlying causes and establish strategies to prevent similar outcomes in future PDCs. Resolving these challenges will be pivotal in unlocking the full therapeutic potential of this emerging class of targeted therapies.

Although the FDA has withdrawn approval for Pepaxto, the EMA and the UK's MHRA have approved it under the brand name Pepaxti for use in combination with dexamethasone to treat relapsed or refractory multiple myeloma [36, 37]. The EMA concluded that Pepaxti's benefits outweigh its risks, granting it full approval on 18 August 2022 [36], followed by MHRA authorisation on 11 November 2022 [37]. Both decisions were informed by overall survival data from the large OCEAN (Phase III) study across relevant patient groups.

#### 5.1.2 CBX‐12

**FIGURE 3:**
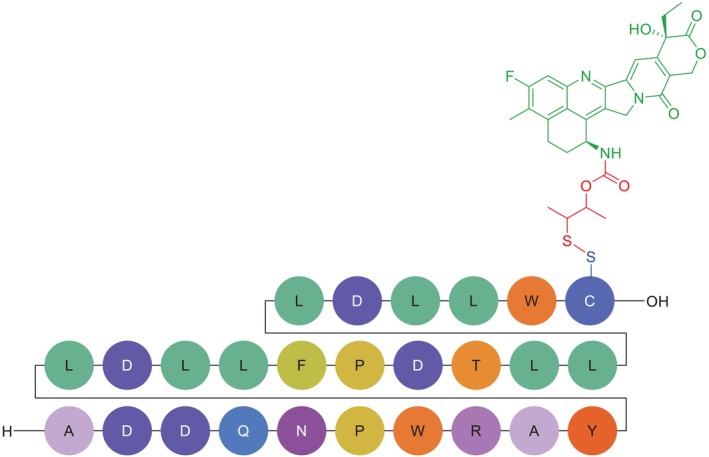
Chemical structure of CBX‐12. Green, exatecan payload; red, self‐immolating, glutathione sensitive linker; blue, homing peptide linking point; black, *C*‐terminal.

The clinical development of CBX‐12 (Table 2) represents a significant innovation in PDC therapeutics, demonstrating that receptor‐independent targeting can achieve potent antitumour activity [41]. This 26‐mer conjugate, developed by Cybrexa Therapeutics, has successfully transitioned from preclinical evaluation to clinical trials based on its unique mechanism of action and compelling therapeutic profile. Preclinical characterisation of CBX‐12 revealed significant tumour growth inhibition across multiple models, favourable biodistribution and tumour‐selective accumulation and an exceptional safety window with minimal off‐target effects. These findings supported FDA clearance of its Investigational New Drug (IND) application, with Phase I trials (NCT06315491) completing enrolment on 16 September 2024 (Figure 3). The observed clinical activity in platinum‐resistant ovarian cancer patients—particularly in those with receptor‐negative tumours—prompted the immediate initiation of Phase II evaluation on 7 October 2024 [41]. The success of CBX‐12 challenges the prevailing dogma that targeted therapies require overexpressed receptors, offering new hope for patients with traditionally ‘undruggable’ cancers. Its progress through the clinical development pipeline marks an important milestone in the evolution of precision oncology therapeutics.

**TABLE 2:** PDCs in the pipeline.

No.	Name	Homing peptide	Receptor	Linker	Payload	Indication	Route of administration and frequency	Company	Phase	Ref.
1	AEZS‐108^a^ (terminated)	D‐Lys6 GHRH	GnRH‐R	Amide	DOX	Endometrial and ovarian cancer	IV 2‐h infusion every 21 days	AEterna Zentaris	III	[38]
2	ANG1005	Angiopep‐2	LRP1	Ester	PTX	Brain metastases from breast cancer	IV infusion 600 mg/m^2^ every 3 weeks	Angiochem Inc	III	[39]
3	BT1718	Bicyclic peptide	MT1‐MMP	Disulfide	DM1	Solid tumours	IV infusion 3 or 10 mg/kg twice weekly	Bicycle Therapeutics	II	[20]
4	BT8009	Bicyclic peptide	Nectin‐4	Amide	MMAE	Advanced or metastatic urothelial cancer	IV 15‐min infusion once weekly infusion for 5 doses over 32 days	Bicycle Therapeutics	III	[40]
5	CBX‐12	Alphalex	Not applicable	Amide	Exetecan	Solid tumours, platinum‐resistant and refractory ovarian cancer	IV infusion once weekly every 28 days	Cybrexa Therapeutics	II	[41]
6	CBP‐1008	CB‐20BK	FRα, TRPV6	Amide	MMAE	Solid tumour	IV infusion every 2 weeks	Coherent Biopharma	I	[42]
7	CBP‐1018	LDC10B	FOLR1, PSMA	Amide	MMAE	Lung tumour	IV infusion once weekly for 4 weeks	Coherent Biopharma	I	[20]
8	G‐202	DγEγEγEγE	PSMA	Amide	Thapsigargin	Heptocellular carcinoma	1‐h IV infusion on Days 1, 2 and 3 for 4 weeks	Gilead Sciences	II	[43]
9	^177^Lu‐PSMA‐617	Glu‐urea‐lysine	PSMA	Nal and TXA	^177^Lu with DOTA chelator	Prostate cancer	IV infusion 7.4 Giga‐becquerel every 6 weeks	Novartis	III	[20]
10	^177^Lu‐Ludotadipep	Ludotadipep	PSMA	Amide	^177^Lu	Prostate cancer	Venous injection every 8 weeks	FutureChem Co	II	[20]
11	MB1707^b^	CXC	CXCR4	Ester	PTX	Solid tumour	2 IM injections once monthly or every 2 months	Mainline Biosciences	I	[11]
12	NGR015	CNGRCG	pAPN	Amide	hTNF	Malignant pleural mesothelioma	IV infusion 1 h 6.5 mg/kg every 3 weeks	HaploX Biotech	III	[44]
13	PEN‐221	fCYwKTCC	SSTR2	Disulfide	DM1	Lung cancer	IV infusion 1 h every 3 weeks	Tarveda Therapeutics	II	[44]
14	TH1902	TH19P01	SORT1	Amide	DTX	Triple negative breast cancer	1‐h IV infusion weekly	Theratechnologies	I	[45]
15	tTF‐NGR	GNGRAHA	CD13, α_v_β_3_ integrin	Amide	tTF	Soft tissue sarcoma	1‐h IV infusion	Anturec Pharmaceuticals	III	[20]
16	TB511	TAMpep	CD18	GGGGS	dKLA	Advanced solid tumours	Intraperitoneally	Twingpig Biolab	I	[46]

Abbreviations: CXCR4, C‐X‐C chemokine receptor 4; DM1, mertansine; DOX, doxorubicin; DTX, docetaxel; FAP‐a, fibroblast activation protease‐a; FOLR1, folate receptor alpha; FRa, folate receptor‐a; GnRH‐R, gonadotropin‐releasing hormone receptor; hTNF, human tumour necrosis factor; LRP1, low density lipoprotein receptor‐related 1; MMAE, monomethyl auristatin E; MT1‐MMP, membrane Type 1 matrix metalloprotease; pAPN, porcine aminopeptidase N; PSMA, prostate specific membrane antigen; PTX, paclitaxel; SORT1, sortillin; SSTR2, somatostatin receptor 2; TRPV6, transient receptor vanilloid subfamily member 6; tTF, truncated tissue factor.

^a^ AEZS‐108, the study was terminated because it did not demonstrate significant improvement in progression‐free survival (PFS) compared with doxorubicin alone [47].

^b^ MB1707, the study was withdrawn due to a lack of supporting data for efficacy, safety and commercial viability.

**FIGURE 4:**
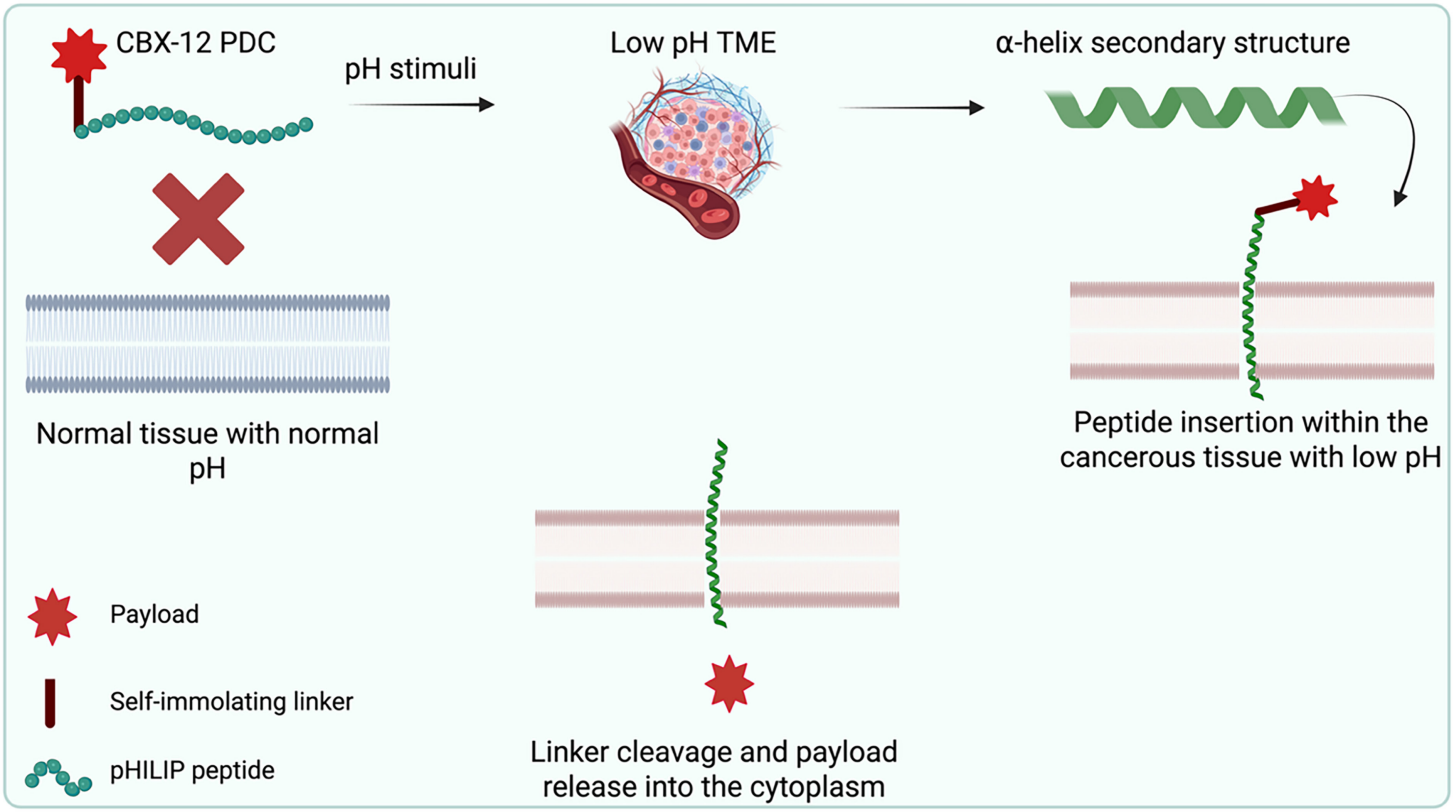
CBX‐12 mechanism of action. TME, tumour microenvironment.

CBX‐12 exemplifies an innovative PDC design that capitalises on fundamental differences between tumour and normal cell physiology. Its structure comprises a pH‐low insertion peptide (pHLIP; alphalex) as targeting moiety, a glutathione‐sensitive, self‐immolating spacer as linker and the potent topoisomerase I inhibitor exatecan as payload (Figure 4) [32]. The alphalex peptide mediates tumour‐specific delivery through a pH‐dependent structural transition, where in acidic TMEs (pH 6.5–7.4) [48, 49], maintained by Warburg metabolism [27], alphalex adopts an α‐helical conformation that inserts directly into plasma membranes [32]. On the other hand, at physiological pH (7.4) of healthy tissues, the peptide remains unstructured, preventing membrane interaction [32]. This differential targeting provides an intrinsic safety mechanism, as the conjugate remains inert in normal tissues until encountering acidic tumour conditions [32].

TOP1 inhibitors are highly potent DNA‐damaging agents that induce cancer cell death by blocking DNA relaxation, thereby obstructing replication [50]. Their integration into PDCs has gained momentum, building on their established role in ADCs [51]. The strategic incorporation of exatecan as a TOP1 inhibitor into PDCs, exemplified by CBX‐12, underscores a paradigm shift in targeted therapy. Unlike ADCs, PDCs leverage pH‐sensitive insertion peptides (pHLIPs) to bypass antigen reliance, enabling tumour‐selective delivery independent of surface markers [32]. This approach not only overcomes ADC limitations but also expands applicability across diverse cancer types. By combining TOP1 inhibitors with pHLIP technology, PDCs like CBX‐12 demonstrate enhanced precision and the potential to exceed current clinical outcomes, paving the way for more inclusive and effective treatments [32].

The development of TOP1 inhibitor‐based PDCs was driven by the need to address the dose‐limiting toxicities and myelosuppression observed with free exatecan administration in clinical settings [31]. Preclinical evaluation by Gayle and coworkers demonstrated CBX‐12’s superior safety profile compared with unconjugated exatecan, showing significantly reduced peripheral toxicity while maintaining antitumour efficacy [32]. These promising results translated to Phase I clinical trials, where CBX‐12 successfully replicated its improved therapeutic index [41]. The compound's progression through Phase II trials further underscores its potential to redefine treatment paradigms by delivering enhanced efficacy with reduced systemic toxicity, marking a significant advancement in targeted TOP1 inhibitor delivery.

### 5.2 Receptor‐Dependent Homing Peptide Paradigm

Receptor overexpression on cancer cell membranes offers a valuable opportunity for targeted therapies, PDCs. The high‐affinity binding of homing peptides to these receptors enhances tumour specificity and therapeutic efficacy, directly improving patient outcomes [24]. However, selecting the optimal homing peptide is critical—not only to maximise binding affinity but also to leverage favourable physicochemical properties that enhance conjugate stability [52]. As a result, thorough evaluation of homing peptides is essential in assessing the targeting potential and overall effectiveness of PDCs in cancer treatment [52]. By incorporating a CTP into PDC design, these conjugates achieve high‐affinity receptor engagement, significantly improving tumour selectivity and therapeutic precision [52]. This strategy capitalises on tumour‐specific molecular signatures while minimising off‐target effects, representing a promising advancement in oncology drug development [52].

In this review, we will explore key receptors implicated in tumour progression, their therapeutic relevance and strategies to selectively engage them using peptides as targeting moieties to enhance drug specificity and efficacy.

#### 5.2.1 Somatostatin Receptor–Targeting PDC

Somatostatin receptors (SSTRs), a family of five G‐protein coupled receptor (GPCR) subtypes [10], play key regulatory roles in cell signalling by modulating hormone secretion and growth factor production [53]. Their ability to inhibit proliferation is particularly relevant in NETs, as well as in breast and lung cancers [53]. The frequent overexpression of SSTRs across these malignancies makes them ideal molecular targets for PDCs, enabling tumour‐selective delivery of cytotoxic agents while sparing healthy tissues [53].

**FIGURE 5:**
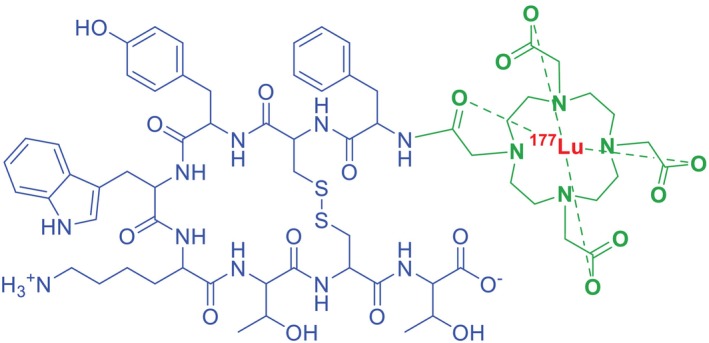
Chemical structure of ^177^Lu‐DOTA‐TATE (Lutathera). Green, chelator; red, radionuclide; blue, somatostatin receptor peptide (octreotate).

The somatostatin receptor‐targeting peptide Tyr3‐octreotate (TATE) serves as the homing peptide in the FDA‐approved peptide‐drug conjugate Lutathera (^177^Lu‐DOTA‐TATE), where it facilitates targeted delivery of the β‐emitting radionuclide ^177^Lu to tumours overexpressing SSTRs [10]. This PDC utilises the chelator DOTA (tetraazacyclododecane‐tetraacetic acid) to stably conjugate the radioactive payload, enabling precise cancer cell targeting while minimising off‐target effects (Figure 5).

Following binding to SSTRs, the ^177^Lu‐DOTA‐TATE complex is internalised via endocytosis, delivering high‐energy β radiation that induces single‐ and double‐stranded DNA breaks, ultimately triggering apoptotic cancer cell death [14]. Although Lutathera (^177^Lu‐DOTA‐TATE) effectively achieves its primary therapeutic goal, its intravenous administration is associated with side effects, including myelosuppression (reduced blood cell counts), elevated liver enzymes, nausea, vomiting, hyperglycaemia and hypokalaemia [13, 54]. These adverse events underscore the need for careful patient monitoring during treatment.

Approved on 26 January 2018 and manufactured by Novartis Pharmaceuticals [2, 55], Lutathera remains the only currently marketed PDC [12]. The TATE homing peptides' high affinity for SSTRs, particularly Subtype 2, exemplifies the potential of receptor‐targeted PDCs in precision oncology, combining tumour‐selective delivery with potent radiation therapy for treating NETs [56]. This successful clinical application demonstrates how strategic homing peptide selection and stable payload conjugation can overcome historical challenges in PDC development [13].

#### 5.2.2 Gonadotropin‐Releasing Hormone Receptor (GnRH‐R)–Targeting PDC

The GnRH‐R family comprises two subtypes, GnRH‐R1 and GnRH‐R2, both belonging to the G protein‐coupled receptor (GPCR) superfamily [10]. GnRH‐R1 is notably upregulated in reproductive tissues and the pituitary gland across multiple cancer types, including breast and prostate cancer [57]. This overexpression, combined with the inherent antiproliferative effects of GnRH‐R1 analogues, makes it an attractive target for PDCs in cancer therapy [58]. The high binding affinity of PDCs for GnRH‐R1 enhances tumour‐selective drug delivery, reinforcing its potential as a precision oncology strategy. The GnRH‐R2 receptor is widely considered a pseudogene in humans and numerous other mammalian species, casting doubt on its functional relevance [59]. This classification raises significant questions about its potential role in cancer therapeutics, as current evidence does not support its existence as a functional receptor [60]. Consequently, therapeutic strategies targeting GnRH‐R2 may require re‐evaluation in light of these genetic findings. Further molecular characterisation is needed to definitively resolve this biological uncertainty and assess whether any residual receptor functionality might exist.

Several GnRH‐R–targeting PDCs are advancing through clinical development, among which AEZS‐108 has emerged as a particularly promising therapeutic candidate [38]. This conjugate (Table 2) employs a synthetic D‐Lys6‐modified GnRH analogue as its homing moiety, engineered for enhanced binding affinity to GnRH‐R—a receptor overexpressed in approximately 80% of endometrial carcinomas [61]. In a Phase II clinical trial conducted by Emons et al., 43 patients received AEZS‐108 as a 2‐h intravenous infusion on Day 1 of a 21‐day cycle for 6–8 cycles [38]. The treatment demonstrated clinically meaningful activity, with 5% of patients achieving complete remission, 18% showing partial remission and 44% maintaining disease stabilisation for ≥ 6 weeks—outcomes that collectively validate both the targeting strategy and therapeutic mechanism [38]. However, observed hematologic toxicities (including four cases of grade 3/4 neutropenia and leukopenia) highlight the need for additional optimisation of the treatment regimen. These findings have prompted further investigation in Phase III trials aimed at refining the dosing schedule to improve the therapeutic index—specifically seeking to enhance antitumour efficacy while mitigating treatment‐related myelosuppression [38]. The ongoing clinical evaluation of AEZS‐108 continues to provide critical insights into the balance between potency and safety in receptor‐targeted PDC therapies.

#### 5.2.3 Epidermal Growth Factor Receptor (EGFR)–Targeting PDC

EGFRs, a subclass of receptor tyrosine kinases, play a pivotal role in oncogenesis through their tyrosine phosphorylation‐mediated activation of cell signalling cascades that drive uncontrolled cellular proliferation [62]. The frequent overexpression of EGFRs across multiple cancer types—including breast, colorectal and gastric carcinomas—positions them as critical molecular targets for next‐generation therapies such as PDCs [63]. Particularly noteworthy is human epidermal growth factor receptor 2 (HER2), an EGFR family member whose overexpression correlates with highly aggressive tumour phenotypes and poor clinical outcomes, making it an especially compelling target for precision oncology approaches [64]. This molecular signature not only validates EGFR/HER2 as biologically relevant targets but also underscores their potential for exploitation in PDC‐based therapeutic strategies aimed at delivering cytotoxic payloads specifically to malignant cells while sparing normal tissues [64].

**FIGURE 6:**
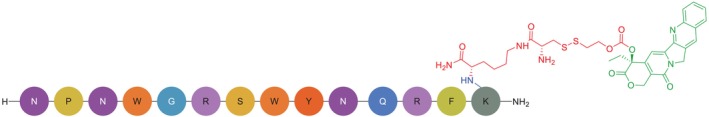
Chemical structure of CPTZ8. Green, Camptothecin payload; red, amide linker; blue, homing peptide linking point; black, *C*‐terminal.

A comprehensive study by Wu and coworkers systematically evaluated 12 distinct PDCs incorporating a Camptothecin payload linked to the HER2‐homing peptide, NPNWGRSWYNQRFK [64]. Among these candidates, CPTZ8 (Figure 6) demonstrated superior HER2‐binding affinity, as validated by surface plasmon resonance assays and competitive binding studies in HER2‐positive (SK‐BR‐3) and HER2‐negative (MDA‐MB‐231) breast cancer cell lines [64]. The selective targeting capability of CPTZ8 underscores its potential to minimise off‐target toxicity while delivering cytotoxic payloads specifically to HER2‐overexpressing tumours [51].

Further in vivo studies in SK‐BR‐3 tumour‐bearing nude mice reinforced CTPZ8's HER2‐targeting specificity and demonstrated enhanced antiproliferative effects compared with free Camptothecin, while maintaining a favourable biosafety profile [64]. Notably, CPTZ8 exhibited reduced haemolytic risk—a critical advantage over unconjugated Camptothecin, which is limited by systemic toxicity [64]. Although this Camptothecin‐based PDC has yet to advance to interventional clinical trials, its dual promise of efficacy and safety in preclinical models positions CPTZ8 as a strong candidate for future translational development [64]. These findings underscore the potential of HER2‐directed PDCs to improve therapeutic indices in aggressive breast cancers [64].

#### 5.2.4 Integrin‐Targeting PDC

Integrins are transmembrane heterodimers consisting of alpha and beta subunits, which contribute to the migration, proliferation and survival of cancer cells [65]. A beneficial attribute of integrins includes not only their diagnostic function due to their relevant overexpression but also their ability to demonstrate dynamic linkage between the extracellular matrix and the cytoplasm of cancer cells [65]. This highlights the potential of integrins in mediating proliferation, metastasis and apoptosis, which enhances prospects for cancer therapeutics [65].

To date, 18 alpha and eight beta chains exist, combining in different formations to represent 24 integrin receptors [66]. This defines contingency for experimentation with different integrins to investigate which are the most valuable in cancer cell targeting. For example, analysis of integrin αvβ6 highlights 85% expression on pancreatic cancer cells, including pancreatic ductal adenocarcinoma (PDAC) [33].

The SG3299 PDC, developed by Spirogen Ltd, incorporates an arginine–glycine–aspartate (RGD)–based homing peptide (A20FMDV2) engineered for high‐affinity targeting of integrin αvβ6—a cell adhesion receptor overexpressed in aggressive carcinomas [67]. Moore and colleagues conducted in vitro studies evaluating the tumour‐targeting specificity of SG3299 through comparative growth inhibition assays in αvβ6‐positive versus αvβ6‐negative cell lines [67]. The results demonstrated remarkably selective cytotoxicity, with αvβ6‐expressing cells showing up to 78‐fold greater sensitivity to SG3299 at equivalent doses [67]. This compelling differential activity provides robust validation of the PDC's targeting mechanism, confirming efficient RGD‐mediated delivery of cytotoxic payload specifically to integrin αvβ6‐positive cancer cells while sparing normal cells [67]. The study's findings significantly advance the field of integrin‐targeted therapies by establishing SG3299 as a promising candidate that overcomes the historical limitations of non‐specific RGD agents [67]. These results not only demonstrate the potential for tumour‐selective cancer cell eradication but also suggest the possibility of safer therapeutic windows compared with conventional chemotherapy [67]. Although these in vitro data are highly encouraging, further in vivo pharmacokinetic and pharmacodynamic studies would strengthen the case for clinical translation of this targeted therapeutic approach [67].

Additionally, exploration of SG3299 PDC was conducted in a human xenograft model [67]. The cytotoxic warhead A20FMDV2 was conjugated to a molecularly specific vector VP1 coat protein of foot‐and‐mouth disease virus (FMDV) [67]. This enhancement in specific targeting towards integrin αvβ6 was shown to increase the survival rate of mice, and the results were indicative of tumour regression, re‐emphasising SG3299 PDC potential. Furthermore, immunohistochemistry studies were conducted to highlight how SG3299 PDC induced DNA damage and cancer cell apoptosis [67]. This mechanism supplements the evidence for increased toxicity evident in positive cell lines and improved survival rate of mice, which reinforces the opportunity for integrin‐targeted cancer therapeutics in the treatment of PDAC [67].

Emerging evidence from Brown and colleagues challenges the translational relevance of conventional xenograft models for evaluating RGD‐targeted therapies in PDAC [34]. The PDAC TME presents unique therapeutic hurdles, characterised by its hypoxic, nutrient‐deprived and acidic conditions coupled with strong immunosuppressive elements [68]. These hostile features critically compromise RGD peptide efficacy through multiple mechanisms: impaired drug penetration due to fibrotic stroma, dynamic modulation of integrin expression patterns and activation of resistance pathways via immunosuppressive cells [34]. This stark contrast between controlled experimental models and the complex clinical reality of PDAC underscores a fundamental limitation in current PDC development paradigms [34]. For meaningful progress, future research must prioritise advanced model systems that better recapitulate human TME complexity, including patient‐derived organoids and engineered stromal cocultures, while exploring combinatorial approaches to overcome these microenvironmental barriers. Although integrin‐targeting PDCs demonstrate compelling mechanistic rationale, their ultimate clinical utility in PDAC will depend on addressing these TME‐imposed challenges [34].

Recent advances in immunocompetent mouse models of PDAC have helped address previous limitations by more accurately recapitulating the immunosuppressive microenvironment and clinical features of human disease. Researchers first established tumours using integrin αvβ6‐positive cell lines, with bioluminescence imaging confirming successful engraftment 7 days postinoculation [34]. Treatment with the targeted SG3299 PDC (20‐μg/kg payload equivalent) demonstrated significant therapeutic advantages: Treated mice exhibited extended median survival of 48 days compared with just 26 days for nontargeting PDC controls [34]. Furthermore, SG3299‐treated animals showed markedly less body weight loss—a key indicator of improved tolerability and reduced disease burden [34]. These findings provide compelling evidence that SG3299 maintains its efficacy even in a more clinically relevant PDAC model that incorporates critical elements of the human TME [34]. The results suggest that targeted integrin αvβ6 delivery can overcome some of the barriers posed by the immunosuppressive PDAC niche, offering renewed promise for this therapeutic approach. Importantly, the preserved therapeutic window (evidenced by reduced weight loss) underscores the potential clinical translatability of this targeted strategy [34].

The immunocompetent mouse PDAC model, while confirming the survival benefit observed in human xenograft studies, revealed an important therapeutic limitation: Neither model demonstrated actual tumour regression [34]. This finding suggests that although SG3299 PDC shows enhanced efficacy in early‐stage disease, its effectiveness diminishes as the complex TME becomes established [34]. The contrast between these in vivo results and more promising in vitro data from Moore's study highlights the TME's critical role in limiting PDC performance in PDAC [34]. These observations emphasise three key points: First, current preclinical models must better incorporate TME complexity to accurately predict clinical outcomes; second, existing integrin‐targeting approaches may need modification to overcome microenvironmental barriers; and third, there is a pressing need for innovative strategies to enhance PDC activity within established tumours [34]. Moving forward, the field must focus on developing TME‐adapted homing peptides, exploring combination therapies with stroma‐modifying agents and creating more sophisticated preclinical models that capture tumour–TME dynamics. Only through such advances can researchers fully realise the clinical potential of integrin‐targeted PDCs for PDAC treatment [34]. For more in‐depth analysis of integrins as targets, readers are referred to an excellent review by Paulus and Sewald [66].

## 6 Linkers

Although homing peptide selection is vital in determining the target for PDC, linker selection is critical as linker structural integrity and timing of payload release determine the circulation exposure of the drug in vivo [26]. This emphasises their importance for adequate drug delivery to the target site. Ideally, cleavage of the linker by proteolytic enzymes would occur once the PDC has reached its target to ensure maximum cellular uptake of the therapeutic agent [2]. However, premature cleavage of PDC is a limitation within PDC development due to the consequence of the ubiquitous overexpression of various proteolytic enzymes within circulation [20]. Therefore, attentive linker selection essentially should be chosen based on how the functional groups of the linker molecule act in vivo or behave intracellularly after PDC uptake [69].

The linker's solubility profile critically impacts PDC efficacy by influencing systemic distribution and clearance kinetics. Since peptides undergo rapid renal filtration because of their small size (2–20 kDa), optimal linker hydrophilicity must balance sufficient plasma exposure with effective tumour penetration. This careful optimisation enhances the therapeutic window by improving tumour accumulation while minimising off‐target effects—a key consideration for clinical translation [20, 69].

The critical role of linker chemistry in PDC optimisation was systematically demonstrated by Liang et al., who compared disulfide (SS), thioether (S) and Val‐Cit (VC) linkers in αvβ3‐targeted doxorubicin conjugates [70]. Their work revealed how linker selection governs cellular uptake specificity, subcellular trafficking patterns and therapeutic efficacy—providing a framework for rational linker design in PDCs [70].

**FIGURE 7:**
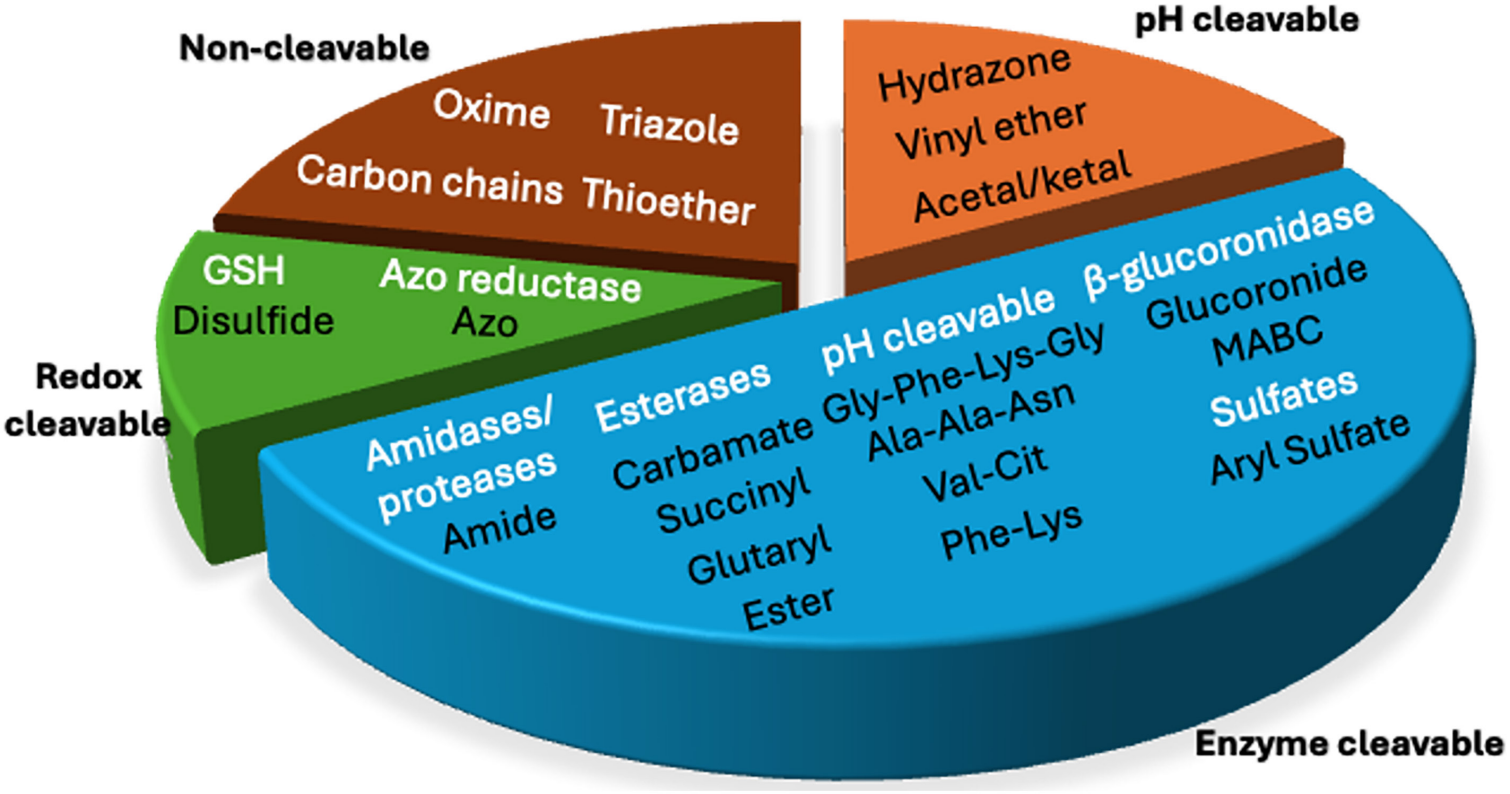
Schematic representation of common linkers used in PDCs and their chemistry.

Examples of common linker alternatives include cleavable linkers (PH‐sensitive, enzyme‐cleavable, redox‐sensitive) and noncleavable linkers (Figure 7) [71].

### 6.1 Enzyme‐Cleavable Linkers

Enzyme‐cleavable linkers represent a critical component of PDCs, designed to undergo enzymatic hydrolysis upon reaching target cells to facilitate precise payload release [71, 72]. These linkers demonstrate remarkable cleavage specificity, as evidenced by their successful implementation in next‐generation PDCs like BT8009 and CBP1008 (Table 2). However, their clinical translation requires thorough evaluation of cleavage stability during systemic circulation. A key challenge lies in balancing the linker's sensitivity to target‐associated enzymes with its resistance to premature cleavage by plasma enzymes or non‐specific proteases [20, 69]. This stability profile directly impacts both therapeutic efficacy (by ensuring sufficient intact conjugate reaches tumour sites) and safety (by minimising off‐target payload release) [73]. Current research efforts must therefore focus on comprehensive pharmacokinetic assessments to quantify (1) circulatory half‐life of intact conjugates, (2) rates of premature linker cleavage and (3) correlation between cleavage patterns and therapeutic outcomes [26]. Addressing these parameters will be essential for optimising enzyme‐cleavable linkers and advancing PDCs through regulatory approval processes [11]. Advanced linker technologies that ensure plasma stability while enabling tumour‐specific activation—as demonstrated by CBX‐12’s pH/glutathione‐responsive design—are critical for optimising PDC efficacy and safety [32].

#### 6.1.1 Amide and Ester Bond Linkers

**FIGURE 8:**
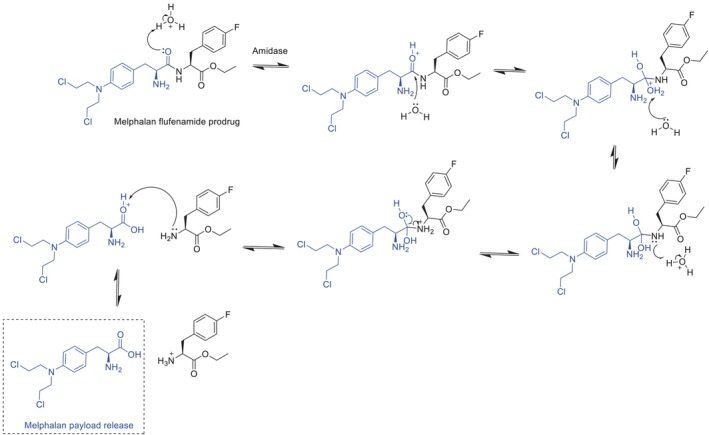
Acidic hydrolysis mechanism of linker cleavage in Pepaxto via amidase enzymes. Amidase, aminopeptidase N (APN) also known as CD13.

Selection of amide linkers stems from their unique dual characteristics: They demonstrate remarkable stability during systemic circulation while remaining highly susceptible to enzymatic cleavage within the TME [74]. This controlled release mechanism occurs primarily in endosomal/lysosomal compartments, where elevated amidase concentrations efficiently hydrolyse the bond [11, 75]. Pepaxto utilises an amide bond to tether its melphalan payload to the lipophilic, peptide‐inspired amide‐based drug (homing) composed of melphalan and p‐fluoro‐L‐phenylalanine [75]. The amide bond's predictable cleavage behaviour under acidic pH conditions and enzymatic exposure makes it particularly valuable for ensuring targeted payload release while minimising premature drug detachment (Figure 8) [24]. These pharmacodynamic advantages explain why amide linkers continue to be a cornerstone of PDC design, though ongoing research seeks to further optimise their stability profile through structural modifications and protective strategies.

As illustrated in Figure 8, although most aminopeptidases hydrolyse *N*‐terminal amino acids from peptides, they exhibit distinct substrate specificity with preferential cleavage of neutral amino acids [76]. Amide linkers offer significant advantages for PDC design; however, their clinical application faces a critical challenge: maintaining sufficient systemic stability during circulation prior to reaching target sites. Despite their relative resistance to hydrolysis in blood plasma, amide bonds remain vulnerable to premature cleavage by circulating amidases and non‐specific proteases, potentially leading to off‐target payload release and reduced therapeutic efficacy. This limitation was particularly evident in Pepaxto's clinical performance, where suboptimal linker stability may have contributed to its eventual market withdrawal.

The pharmacokinetic paradox of amide linkers—requiring both circulatory stability and rapid tumoral activation—presents an ongoing optimisation challenge for PDC developers [11]. Current strategies to address this include (1) structural modifications like β‐amino acid substitutions to sterically hinder premature enzymatic access, (2) incorporation of self‐immolative spacers that require sequential activation steps and (3) development of microenvironment‐responsive shielding groups that protect the amide bond until tumour localisation [11, 24]. These innovations aim to preserve amide linkers' advantageous cleavage properties while overcoming their circulatory stability limitations, potentially unlocking their full potential for next‐generation PDCs [11].

Recent advances in PDC design have highlighted the growing utility of ester bonds as complementary linkers to traditional amide bonds, particularly for targeting enzyme‐rich lysosomal and endosomal compartments. A pivotal study by Karampelas and colleagues demonstrated this approach through the development of a dual‐linker PDC for advanced prostate cancer, combining both ester and amide chemistries in a single conjugate [35]. The conjugate features a gemcitabine payload connected via an ester bond to a glutaryl spacer, which in turn forms an amide bond with the [D‐Lys6]‐GnRH targeting peptide [35]. This innovative architecture capitalises on the distinct advantages of each linker type: The amide bond provides necessary stability during systemic circulation, whereas the ester bond enables rapid payload release upon encountering intracellular esterases [35]. The design specifically addresses the challenge of achieving both circulatory stability and efficient tumour‐specific activation, leveraging the differential expression of esterases between plasma and target cells [35].

By employing the glutaryl spacer as a molecular bridge between these two cleavage mechanisms, the system allows for sequential drug release—first, through amide bond hydrolysis in the TME, followed by esterase‐mediated payload liberation [35]. This work not only demonstrates the potential of ester bonds in PDC development but also establishes a framework for optimising linker combinations to balance pharmacokinetic stability with efficient payload delivery [35]. Future studies will need to further characterise the cleavage kinetics of such hybrid systems and evaluate their performance across different cancer types and payload classes [35].

**FIGURE 9:**
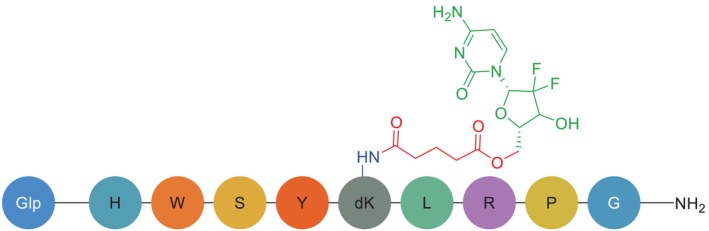
Chemical structure of GSG. Glp, pyroGlu; green, gemcitabine payload; red, ester linker; blue, homing peptide linking point; black, *C*‐terminal.

The research team synthesised and evaluated multiple conjugates through in vitro testing to assess their ability to inhibit proliferation in DU145 and PC3 prostate cancer cell lines [35]. Subsequent analysis revealed that the gemcitabine–succinate–GnRH (GSG) conjugate (Figure 9) emerged as the most effective candidate, demonstrating superior anti‐proliferative activity compared with other tested variants [35].

Building on its demonstrated in vitro efficacy, GSG was advanced to in vivo evaluation using a xenograft mouse model because of its high potency, enhanced stability and favourable pharmacokinetic profile [35]. The study revealed several key findings: (1) GSG administration significantly suppressed tumour growth compared with saline controls, validating the conjugate's in vivo stability and the glutaryl linker's effectiveness; (2) GSG achieved comparable antitumour effects at a dramatically reduced dose (18.8 μmol/kg) relative to free gemcitabine (454.5 μmol/kg), representing a 24‐fold decrease in required dosage; and (3) this dose‐sparing effect suggests GSG may substantially improve the therapeutic window by maintaining efficacy while potentially reducing the systemic toxicity associated with conventional gemcitabine chemotherapy [35].

These results collectively demonstrate GSG's dual advantage as both a more potent and potentially safer therapeutic option, with its targeted delivery mechanism enabling precise tumour localisation while minimising off‐target effects [35]. The data provide compelling evidence for GSG's clinical potential in prostate cancer treatment, particularly in addressing gemcitabine's historical challenges of rapid metabolism and dose‐limiting toxicities [35]. Further studies should focus on comprehensive toxicity profiling and potential combination strategies to maximise GSG's therapeutic utility [35].

Although the exact mechanism underlying GSG's enhanced efficacy remains under investigation, pharmacokinetic analyses revealed significantly higher plasma concentrations of intact gemcitabine and correspondingly lower levels of its inactive metabolite 2′,2′‐difluoro‐2′‐deoxyuridine (dFdU) following GSG administration compared with equivalent doses of free gemcitabine [35, 58]. These findings strongly suggest that the conjugate's design confers improved metabolic stability, thereby preserving therapeutic payload availability [35]. However, Alas and coworkers have proposed an alternative interpretation, suggesting that elevated gemcitabine levels might instead reflect premature linker cleavage rather than true stabilisation [71]. This perspective currently lacks substantive experimental support, as no studies have systematically evaluated dose‐escalation strategies to account for potential payload loss. Importantly, the available evidence—including GSG's demonstrated tumour growth inhibition at markedly reduced doses—more robustly supports the stability and efficacy hypothesis [35]. The conjugate's performance metrics (enhanced antitumour activity with dose‐sparing effects) align better with a model of targeted delivery and controlled release rather than non‐specific premature activation. Further mechanistic studies incorporating tracer‐labelled conjugates and detailed metabolite profiling could help resolve this scientific discourse while optimising next‐generation PDC designs.

The potential instability of PDC linkers presents a complex challenge, with carboxylesterase (ce) enzymes emerging as particularly problematic due to their ability to hydrolyse amide, ester and carbamate bonds [77]. This concern is amplified by interspecies differences, as Nagy and colleagues demonstrated that mouse plasma exhibits 10‐fold higher ce activity than human plasma [78], potentially skewing preclinical results. To address this translational gap, Dorywalska and coworkers conducted stability studies in cynomolgus monkeys—a model with human‐like ce expression—and observed significantly reduced linker degradation compared with murine models [78]. These findings suggest that PDCs may demonstrate enhanced stability in clinical settings, potentially improving patient outcomes [78]. However, the precise mechanisms underlying this residual instability remain unclear, with possibilities ranging from low‐level proteolytic activity to endosomal recycling processes. This uncertainty highlights two critical barriers in PDC development: (1) the difficulty in establishing truly predictive efficacy models that accurately replicate human plasma conditions and (2) the need for advanced analytical methods to distinguish between enzymatic and nonenzymatic degradation pathways [78]. Overcoming these challenges through targeted research—particularly in optimising linker chemistry for human physiology—will be essential for successful translation of PDCs from preclinical studies to interventional clinical trials [78].

The research by Karampelas and colleagues provides promising evidence for the therapeutic potential of PDCs in prostate cancer, with their GSG conjugate demonstrating both enhanced tumour cell suppression and improved metabolic stability in vitro [77]. However, significant translational challenges persist, particularly regarding linker stability across preclinical models [77]. Although initial murine studies revealed instability concerns, subsequent primate experiments using cynomolgus monkeys—which more closely mimic human CE activity—showed markedly improved stability profiles, underscoring the limitations of current preclinical models in predicting clinical performance [72, 77]. These conflicting results emphasise the critical need for more human‐relevant testing systems and a deeper understanding of the fundamental mechanisms governing PDC efficacy and stability [77].

The field particularly lacks comprehensive studies on receptor‐targeted gemcitabine conjugates and optimal linker design strategies that can maintain circulatory stability while ensuring efficient tumour‐specific payload release. Addressing these knowledge gaps through systematic investigation of linker chemistry, detailed pharmacokinetic profiling in advanced models and development of strategies to prevent premature cleavage will be essential for translating promising PDC candidates like GSG into clinically viable therapies. Such research must focus not only on overcoming technical hurdles but also on establishing standardised approaches to evaluate and optimise these complex therapeutic agents for prostate cancer treatment [72].

#### 6.1.2 Carbamate

Sun and colleagues reported CPT‐SSA conjugates JF‐10‐71 and JF‐10‐81, featuring a tuneable carbamate linker for controlled release, which potently inhibit SSTR2‐overexpressing IMR32 neuroblastoma cells while maintaining stability in plasma [79]. Recent studies revealed enhanced efficacy in somatostatin receptor‐positive CA20948 pancreatic cells, surpassing free CPT [80]. In vivo, both conjugates showed dose‐dependent tumour growth inhibition in xenograft models [79]. Although free CPT exhibited stronger cytotoxicity in IMR32 cells (IC50 = 3.1 nM), the conjugates demonstrated a controlled‐release profile (JF‐10‐81: 64.13 nM; JF‐10‐71: 282.50 nM) [79]. Notably, in CA20948 cells (CPT IC50 = 3077 nM), the SSA conjugates were more potent (JF‐10‐81: 1790 nM; JF‐10‐71: 1363 nM). SSA alone showed no activity [79].

The conjugates selectively internalise into SSTR2+ tumours, releasing CPT to block DNA replication [79]. This targeted approach improves specificity, solubility and tolerability over free CPT [79]. Sustained‐release formulations further enhance efficacy with minimal toxicity, highlighting their potential for optimised cancer therapy [79].

#### 6.1.3 Peptides

The dipeptide valine‐citrulline (VC) serves as an enzyme‐cleavable linker that is specifically recognised and cleaved by cathepsin B, a lysosomal carboxypeptidase that is overexpressed in tumour cells [81]. This selective cleavage enables targeted drug release within the lysosomes of cancer cells, enhancing tumour specificity while minimising off‐target effects [81].

Interestingly, Liang and colleagues developed three amphiphilic peptide‐doxorubicin conjugates (APDCs)—cRGD‐SS‐DOX (RSSDOX), cRGD‐S‐DOX (RSDOX) and cRGD‐VC‐DOX (RVCDOX)—using different linker chemistries (disulfide, thioether and Val‐Cit dipeptide) to explore structure–activity relationships (SARs) [70]. These APDCs demonstrated several key advantages: (1) selective targeting of αvβ3 integrin‐positive B16 melanoma cells through receptor‐mediated endocytosis, (2) stimulus‐responsive drug release profiles tailored to each linker's cleavage mechanism and (3) enhanced intracellular drug delivery compared with free doxorubicin [70]. Notably, RSDOX and RVCDOX exhibited superior lysosomal stability and cytoplasmic drug accumulation because of their amide bond hydrolysis in lysosomes, whereas RSSDOX showed reduction‐sensitive release [70]. In vivo studies confirmed these APDCs achieved potent tumour growth inhibition while minimising systemic side effects, highlighting their potential as next‐generation targeted therapies with improved therapeutic windows [70]. The study provides important insights into how linker chemistry influences the pharmacokinetics, biodistribution and efficacy of peptide‐drug conjugates [70].

#### 6.1.4 Disulfide Bond Linkers

Among common linker types, disulfide bonds are especially prone to premature payload release via thiol‐disulfide exchange (e.g., with glutathione, GSH) [82, 83]. Although this may compromise therapeutic efficacy through off‐target drug release, it simultaneously facilitates tumour‐selective activation [82, 83]. Given the stark gradient between high intracellular GSH (millimolar) and low extracellular/plasma GSH (micromolar) concentrations, PDCs remain stable in circulation but are efficiently cleaved within tumour cells, ensuring localised payload release [82, 83].

**FIGURE 10:**
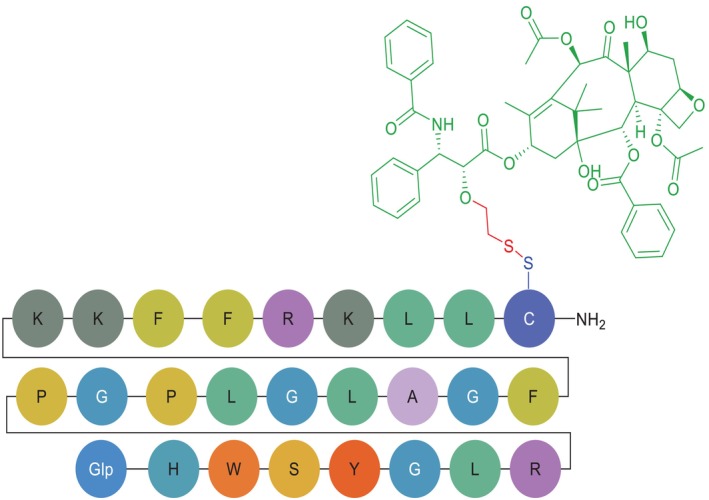
Chemical structure of GSG. Glp, pyroGlu; green, PTX payload; red, disulfide linker; blue, homing peptide linking point; black, *C*‐terminal.

Deng and co‐workers developed a smart drug conjugate in which PTX was linked to a multifunctional peptide containing both a TTP and a CPP (Figure 10) [69].

This TTP‐CPP‐PTX conjugate demonstrated enhanced specificity and efficacy against luteinizing hormone‐releasing hormone (LHRH) receptor‐overexpressing MCF‐7 cells [69]. The conjugate, LTP‐1, exhibited twice the cellular uptake of free PTX and significantly improved cytotoxicity, with an IC50 of 3.8 nM compared with 6.6 nM for PTX alone [69].

### 6.2 Noncleavable Linkers

Noncleavable linkers, such as thioethers, offer significant advantages in PDC design by remaining inert to enzymatic or environmental stimuli in circulation, thereby preventing premature payload release in blood plasma [27]. This inherent stability expands the therapeutic window, allowing for broader dosing flexibility while maintaining a favourable safety profile—a critical consideration in clinical oncology [27, 84]. Furthermore, their structural rigidity does not compromise the homing peptide's ability to engage target receptors, as these linkers demonstrate remarkable conformational flexibility that facilitates optimal binding interactions [27]. By ensuring payload release occurs exclusively upon internalisation and lysosomal degradation of the entire conjugate, noncleavable linkers significantly reduce off‐target toxicity while enhancing tumour‐specific drug delivery [85]. This dual capability—preserving circulatory integrity while enabling efficient intracellular activation—positions noncleavable linker‐based PDCs as a promising strategy to improve the precision and safety of targeted cancer therapies. Their success in ADCs, such as maleimidohexanoyl linker in Blenrep [86], further supports their potential adaptation to PDC platforms, though ongoing research is needed to optimise their application across diverse peptide‐receptor systems.

The use of noncleavable linkers in PDCs presents a critical therapeutic trade‐off regarding the bystander effect. Although these linkers provide superior plasma stability and prevent premature payload release, they inherently limit the payload's ability to diffuse to neighbouring cancer cells following internalisation, as drug liberation occurs only after complete lysosomal degradation of the conjugate [27]. This restriction may reduce efficacy against heterogeneous tumours containing receptor‐negative or poorly accessible cell populations [27]. However, this same characteristic offers potential safety advantages by minimising off‐target toxicity to healthy tissues adjacent to tumours—a significant concern with cleavable linkers that enable extracellular payload diffusion [87].

The constrained biodistribution of noncleavable PDCs may be particularly advantageous when treating cancers near critical anatomical structures or when using highly potent payloads where precise targeting is paramount [27]. This dichotomy highlights the need for context‐dependent linker selection, where the tumour type, microenvironment and payload characteristics dictate the optimal balance between therapeutic coverage and safety [27]. Emerging strategies to address this challenge include the development of TME‐activated linkers and combination approaches with penetration‐enhancing agents, aiming to reconcile the precision of noncleavable platforms with the broader distribution enabled by cleavable systems [27, 87]. The choice between these approaches ultimately depends on carefully weighing the risk–benefit profile for each specific clinical application [27, 87].

The contrasting benefits of cleavable and noncleavable linkers—with the former enabling broader tumour penetration through bystander effects and the latter offering enhanced safety through controlled activation—have spurred interest in hybrid linker systems that combine their advantageous features [87]. Although cleavable linkers currently dominate clinical applications, innovative approaches integrating both linker types are emerging as a promising strategy to optimise the therapeutic index of PDCs [87]. One such development involves coupling a noncleavable thioether linker (formed via Michael addition of a thiol to maleimide) with an amide‐based cleavable component, designed to enable amidase‐mediated payload release [71]. However, this approach faces challenges because of inherent instability caused by retro‐Michael reactions, which can prematurely release thiolate peptides and maleimide moieties prior to tumour targeting—a limitation that currently constrains its therapeutic potential [71].

These findings underscore the critical need for continued research to enhance the stability of hybrid linker systems while preserving their capacity for controlled payload release. Key priorities include developing more robust conjugation chemistries resistant to retro‐Michael decomposition, optimising spacer elements to balance stability and cleavage efficiency and establishing predictive assays to evaluate hybrid linker performance under physiological conditions [71, 87]. Success in this area could yield next‐generation PDCs capable of simultaneously achieving precise targeting, extended circulation stability and optimised tumour‐wide drug distribution—addressing one of the fundamental challenges in targeted cancer therapy [87].

#### 6.2.1 Oxime

Ranđelović et al. developed a series of PDCs derived from the CKAAKN oligopeptide, modified to optimise their therapeutic properties [88]. By replacing cysteine with serine (SKAAKN), they eliminated the non‐essential thiol group, improving conjugate hydrophilicity and solubility while maintaining targeting efficacy [88]. The team attached daunomycin (Dau) via an oxime linkage, ensuring plasma stability while enabling lysosomal release of an active metabolite (Dau = Aoa‐Aaa‐OH) capable of DNA binding—though binding affinity varied with the amino acid (Aaa) moiety [88].

Among the five PDCs tested against PANC‐1 pancreatic cancer cells, Dau = Aoa‐GFLGK (Dau = Aoa)SKAAKN‐OH (Conjugate 4) emerged as the most potent in vitro, achieving > 30% tumour growth inhibition in vivo without the toxicity of free daunomycin [88]. These results highlight the SKAAKN platform as a promising targeted delivery system for pancreatic cancer, either as monotherapy or in combination regimens [88].

#### 6.2.2 Triazole

Zheng and colleagues designed a series of αvβ3‐targeted silicon (IV) phthalocyanines axially conjugated to cyclic RGD peptides through ethylene glycol linkers [89]. The symmetric conjugate 6b, featuring two cRGD ligands, showed remarkable selectivity for αvβ3+ HT‐29 cells, displaying receptor‐dependent cellular uptake (3.8‐fold higher in HT‐29 compared with αvβ3− MCF‐7 cells), enhanced photocytotoxicity (with an IC50 of 0.2 nM for lead compound 2c) and superior ROS generation, as confirmed by integrin‐mediated endocytosis in competitive assays [89]. In vivo studies further demonstrated 6b's efficacy, achieving 75% tumour growth inhibition in H22 tumour models while exhibiting preferential tumour accumulation and no systemic toxicity [89]. These findings highlight 6b as a highly promising tumour‐selective photosensitiser for clinical photodynamic therapy (PDT) [89].

Ke and colleagues synthesised a 1,4‐disubstituted zinc (II) phthalocyanine conjugated with a cyclic Arg‐Gly‐Asp‐D‐Phe‐Lys (cRGDfK) peptide via a triazole linker [90]. Given that cRGDfK is a well‐known αvβ3‐integrin antagonist, this conjugate demonstrated significantly higher cellular uptake in αvβ3(+) U87‐MG cells compared with αvβ3(−) MCF‐7 cells, as confirmed by flow cytometry and fluorescence microscopy [90]. However, despite this selective uptake, the photocytotoxicity of the compound was comparable in both cell lines because of similar intracellular reactive oxygen species (ROS) generation efficiency [90]. Confocal microscopy revealed that the conjugate localised preferentially in the lysosomes of U87‐MG cells [90]. The combination of high selectivity (both at cellular and subcellular levels) and strong photocytotoxicity suggests that this well‐defined conjugate could serve as a promising photosensitiser for targeted PDT.

## 7 Payloads

Highly potent cytotoxic agents are preferentially incorporated into ADCs or PDCs due to two fundamental limitations: (i) Their inherent toxicity prevents standalone administration, or (ii) their therapeutic window requires optimisation to mitigate side effects [24, 91].

**FIGURE 11:**
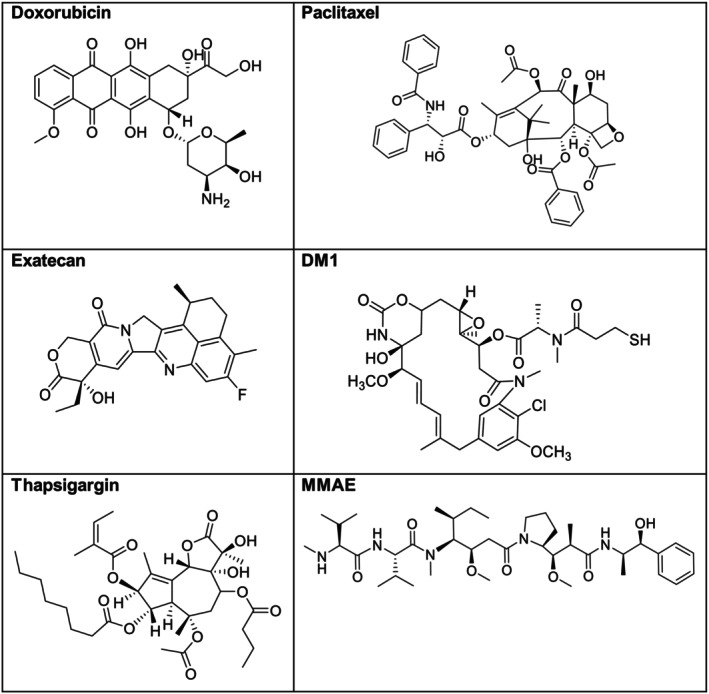
Chemical structures of cytotoxic payloads present in various PDCs.

Diverse chemical structures of payloads employed in PDCs, which typically fall into three classes: (a) ultra‐potent agents (e.g., auristatins), which require targeted delivery because of subnanomolar cytotoxicity and are often derived from natural products such as dolastatin 10 [73, 92, 93]; (b) chemotherapy payloads (e.g., doxorubicin) [94], which benefit from tumour‐selective delivery to reduce off‐target effects and enable dose escalation beyond conventional maximum tolerated dose (MTD) limits; and (c) novel payload classes (e.g., exatecan) [31], which expand mechanisms of action (RNA polymerase II inhibition, TOP1 inhibition), which leverage conjugate platforms to overcome poor pharmacokinetics (Figure 11).

The discovery of novel auristatin payload (PF‐08077285) and drug linker (PF‐08081016), by Moquist and colleagues, initially developed for ADCs, also hold significant promise for PDCs. Given the structural and functional adaptability of auristatins, this payload could be leveraged in PDCs to enhance targeted cancer therapy with potential advantages in tumour penetration, synthesis simplicity and cost‐effectiveness compared with ADCs [95].

## 8 PDC Synthesis

The synthesis of PDCs involves two critical stages: (i) production of individual components (peptides, linkers and payloads) and (ii) their selective conjugation. For peptide synthesis, solid‐phase peptide synthesis (SPPS) remains the gold standard because of its efficiency and scalability [96, 97], though alternative methods like classical solution‐phase synthesis (CSPS), liquid‐phase peptide synthesis (LPPS) [97, 98], native chemical ligation (NCL) [99] and semisynthetic approaches are employed for specific applications [100].

Conjugation chemistry is equally diverse, leveraging multiple strategies to ensure precise and stable linkage: (i) click Chemistry via copper‐catalysed (CuAAC) or strain‐promoted (SPAAC) azide‐alkyne cycloadditions for bioorthogonal coupling [101], or (ii) thiol‐based reactions: maleimide‐thiol or disulfide formation for cysteine‐selective attachment [102], (iii) amide/ester bonds via carbodiimide or active ester‐mediated coupling and (iv) α‐ketoacid‐hydroxylamine (KAHA) ligation: chemoselective KAHA reactions forming stable amide/ester bridges [103]. Moreover, chelation chemistry plays a key role in peptide receptor radionuclide therapy (PRRT), where it is used to create DOTA–peptide conjugates, such as Lutathera [104]. Bugatti has published a comprehensive review that systematically examines the diverse synthetic methodologies and strategic approaches for constructing PDCs [105].

## 9 Computational Software and Artificial Intelligence (AI) Tools

Computational software and AI tools have become indispensable in advancing peptide‐based drug discovery, revolutionising the design of PDCs, antimicrobial peptides, antiviral agents and anticancer therapeutics. Cutting‐edge computational tools now enable sophisticated peptide engineering through multiple approaches: structure‐based design using platforms like Molecular Operating Environment (MOE) [106], deep learning with 3D‐Convolutional Neural Networks [107] and web‐based prediction servers such as GalaxyPepDock [108], I‐TASSER [109, 110], ADMETlab 2.0 [111] and SwissADME [112]. The integration of these complementary computational methods significantly enhances the likelihood of identifying viable therapeutic candidates [113].

Recent breakthroughs in AI‐driven de novo peptide design are particularly transformative. Tools like InSiPS employ parallel genetic algorithms and PPI prediction to engineer synthetic binding proteins (SBPs) with minimised off‐target effects [114], while deep generative models (DGMs) facilitate the creation of novel peptide therapeutics with optimised properties [115]. AI also accelerates drug discovery by enabling high‐throughput virtual screening of extensive compound databases [116], as comprehensively reviewed by Goles et al. [117].

## 10 Conclusion

PDCs have emerged as a promising next‐generation targeted therapy, demonstrating exceptional tumour specificity and favourable safety profiles that position them as strong candidates to build upon the success of ADCs. The field has gained substantial momentum through key milestones, including the FDA approval of Lutathera, the clinical advancement of CBX‐12 and approximately 96 ongoing clinical trials evaluating various PDC candidates. These developments underscore the growing recognition of PDCs' therapeutic potential. However, challenges such as rapid renal clearance, premature linker cleavage and structural instability currently limit their clinical performance, as exemplified by the withdrawal of Pepaxto from the US market. Rather than discouraging further development, these setbacks have highlighted critical areas for optimisation and spurred innovative solutions. CBX‐12 exemplifies a paradigm shift in targeted therapy through its receptor‐independent mechanism, demonstrating how peptide‐based platforms can overcome fundamental limitations of conventional approaches—including antigen heterogeneity, receptor downregulation and sparse target expression in TMEs.

One particularly promising direction involves the use of peptidomimetics like the RGD [118] and RGDF analogues, which offer enhanced metabolic stability, improved target affinity and greater bioavailability compared with native peptides [119]. Such modifications address several key limitations while preserving the fundamental advantages of peptide‐based targeting [119]. The continued evolution of PDC technology—through optimised linker chemistries, advanced payload strategies and innovative peptidomimetic designs—is steadily overcoming early challenges.

With ongoing research focused on improving pharmacokinetic properties and therapeutic indices, PDCs are poised to become an increasingly important class of oncology therapeutics, potentially complementing or surpassing current ADC approaches for certain indications. The field now stands at a critical juncture where translational innovations could unlock the full clinical potential of these targeted agents. The computational paradigm shift—from empirical screening to rational AI‐guided design—promises to overcome historical challenges in peptide therapeutics while unlocking unprecedented precision in targeted drug delivery.

## Conflicts of Interest

The authors declare no conflicts of interest.

## Data Availability

Data sharing not applicable to this article as no datasets were generated or analysed during the current study.
